# Recurrent Thrombotic Thrombocytopenic Purpura Associated With Helicobacter pylori: More Than a Gut Feeling

**DOI:** 10.7759/cureus.88035

**Published:** 2025-07-15

**Authors:** Esperance M Madera, Keon J Sargon, Domonick K Gordon, Alexandra Zodo, Gerarda Corneille, Devon Cole, Gaurav Paul, Ariel S Mandelblum, Daniel Han, Musa Ayyad, Kulsum Farooqi

**Affiliations:** 1 Internal Medicine, Mount Sinai Hospital, Chicago, USA

**Keywords:** adamts13 deficiency, helicobacter pylori, microangiopathic hemolytic anemia (maha), platelet aggregation, thrombotic thrombocytopenic purpura (ttp)

## Abstract

Thrombotic thrombocytopenic purpura (TTP) is a rare, life-threatening hematologic disorder characterized by severe ADAMTS13 deficiency, leading to uncontrolled platelet aggregation, microvascular thrombosis, and multi-organ dysfunction. Although the pathogenesis of immune-mediated TTP (iTTP) is primarily autoimmune, infectious agents, including *Helicobacter pylori* (*H. pylori*), have been implicated as potential triggers. However, the association between *H. pylori *and iTTP remains poorly defined. We describe a case of a 33-year-old male presenting with hematuria, thrombocytopenia, and laboratory evidence of microangiopathic hemolytic anemia. A markedly reduced ADAMTS13 activity and elevated inhibitor level confirmed the diagnosis of iTTP. The patient responded well to plasma exchange and rituximab but experienced relapse following therapy interruption. During the relapse, testing revealed a positive *H. pylori *stool antigen and eradication therapy was initiated. This raised the possibility of *H. pylori *contributing to disease recurrence. This case adds to emerging evidence suggesting a potential role of *H. pylori *in the pathogenesis or exacerbation of iTTP. Proposed mechanisms include molecular mimicry, platelet aggregation via von Willebrand factor interactions, P-selectin expression, and pylori urease, as well as through pro-inflammatory cytokine release. While observational data and isolated case reports have highlighted this association, direct causal links and the therapeutic impact of* H. pylori *eradication in iTTP require further investigation. The recurrence of iTTP in the setting of persistent *H. pylori* infection in this case underscores the need for further investigation into *H. pylori *as a modifiable risk factor in iTTP. Further research is needed to determine if targeted screening and eradication strategies may offer a novel adjunctive approach to reduce recurrence and improve long-term outcomes.

## Introduction

Thrombotic thrombocytopenic purpura (TTP) is a rare but life-threatening hematologic disorder characterized by microvascular thrombosis and severe platelet consumption. The estimated incidence of TTP ranges from two to six cases per million annually [1]. It results from a severe deficiency of ADAMTS13 (a disintegrin and metalloproteinase with a thrombospondin type one motif, member 13), an enzyme responsible for cleaving von Willebrand factor (vWF) multimers. Without treatment, excessive platelet aggregation occurs, leading to microangiopathic hemolytic anemia (MAHA) and multi-organ dysfunction. TTP carries a mortality rate exceeding 90% if left untreated, emphasizing the need for early diagnosis and intervention [2-3]. Although infections, malignancies, and autoimmune diseases are known contributors to TTP, the role of *Helicobacter pylori* (*H. pylori*) remains unclear, and its contribution to pathogenesis is not well understood.

*H. pylori *is primarily recognized for its association with gastric pathology, but emerging evidence suggests that it may also contribute to hematologic disorders. Proposed mechanisms include immune dysregulation, molecular mimicry, and endothelial injury. It has been hypothesized that *H. pylori*-produced cytotoxins, such as cytotoxin-associated gene A (CagA), may contribute to autoimmune dysregulation involving targets such as ADAMTS13; however, this mechanism remains speculative and lacks robust clinical evidence [4]. While the eradication of *H. pylori *has been associated with improved platelet counts in chronic immune thrombocytopenic purpura (cITP), its role in TTP is unconfirmed and supported primarily by isolated case reports [4-5].

This case highlights a potential but underrecognized association between *H. pylori *and recurrent TTP. Although primarily linked to gastric disease, *H. pylori *may contribute to hematologic conditions through mechanisms such as immune dysregulation and endothelial injury. In this 33-year-old male with chronic gastritis and a positive *H. pylori *stool antigen test, the persistent infection raised suspicion for a contributory role in his TTP relapses. While evidence remains limited, this case reinforces the need for further investigation into whether *H. pylori* screening and eradication could reduce TTP recurrence.

## Case presentation

A 33-year-old male with a past medical history of gastritis and crack cocaine use presented to the emergency department with complaints of gross hematuria and dizziness. He reported a three-day history of constant, sharp bilateral flank pain, right groin discomfort, and dysuria characterized by a burning sensation at the end of urination. He also noted hematuria throughout the entire urinary stream without clots, a single episode of emesis containing small blood clots, diaphoresis, and headache. The patient denied fever, chills, shortness of breath, upper respiratory symptoms, chest or abdominal pain, extremity weakness, or fatigue. He stated that his last laboratory testing was performed approximately three years prior and was reportedly normal. He denied a history of hepatitis, HIV (human immunodeficiency virus), autoimmune diseases, intravenous drug use, or prior platelet/blood transfusions.

On physical examination, he was hemodynamically stable. Notable findings included bilateral flank tenderness, scattered purpura on the chest, and mild tenderness in the subscapular region. Initial laboratory results were significant for the following: total bilirubin: 4.9 mg/dL, lactate dehydrogenase (LDH): 1,334 U/L, creatine kinase (CK): 266 U/L, hemoglobin (Hb): 13.6 g/dl, platelets: 8,000/µL, and troponin: 222 ng/L. Urinalysis showed orange-colored urine, protein 600 mg/dL, blood 3+, RBCs >200/hpf, and 10 hyaline casts. Peripheral smear showed severe thrombocytopenia without schistocytes. Hepatitis panel, HIV, and antinuclear antibody (ANA) testing were negative. Table 1 presents a summary of laboratory data with clinical annotations.

**Table 1:** Laboratory data summary with clinical annotations This table summarizes the clinical course of the patient, diagnosed with acquired thrombotic thrombocytopenic purpura (TTP), confirmed by severe ADAMTS13 deficiency and elevated inhibitor levels. It outlines the treatment response to plasma exchange and rituximab, including a temporary interruption (days 7–18) that led to clinical deterioration, followed by partial recovery upon resumption with rituximab and corticosteroids (days 18–21). Additional findings include corrected vitamin B12 deficiency, positive *Helicobacter pylori *infection as a potential trigger, and the absence of disseminated intravascular coagulation (DIC), supported by normal coagulation parameters. Abbreviations: ADAMTS13: a disintegrin and metalloproteinase with thrombospondin type 1 motif, 13; *H. pylori*: *Helicobacter pylori*; DIC: disseminated intravascular coagulation; INR: international normalized ratio; LDH: lactate dehydrogenase; MCHC: mean corpuscular hemoglobin concentration; MCV: mean corpuscular volume; PTT: partial thromboplastin time; RDW: red cell distribution width; WBC: white blood cell

Test	Reference range	Day 1: admission	Day 2	Day 7: post-treatment	Day 18: readmission	Day 21: final discharge	Clinical notes
WBC (×10³/mcL)	4.00–11.00	8.7	9.6	17.3 H	7.3	11.6 H	The elevated WBC on D7 suggests a post-treatment inflammatory response or possible infection. Returns near normal after resuming therapy.
Hemoglobin (g/dL)	13.0–17.0	13.6	11.0	11.4 L	10.7 L	11.0 L	Progressive anemia, likely multifactorial (hemolysis, bone marrow suppression).
Hematocrit (%)	38.6–49.2	37.4 L	39.1	35.5 L	31.1 L	33.8 L	Mirrors Hgb trends; anemia worsens during the treatment gap.
MCV (fL)	80–100	90.4	90.9	100.6 H	100.3 H	103.1 H	Mild macrocytosis could reflect reticulocytosis or vitamin B12 replacement.
MCHC (g/dL)	32.5–35.8	36.4 H	35.9 H	32.2	34.5	N/A	Mildly elevated early, normalizes; possibly spurious or related to hemolysis.
Platelets (×10³/mcL)	150–450	8 L	11 L	243	45 L	68 L	Severe thrombocytopenia initially improves post-plasma exchange. Drops again with missed Rituximab; partially recovers after re-initiation.
RDW (%)	11.9–15.9	12.9	12.8	N/A	17.5 H	17.9 H	Elevated RDW suggests anisocytosis, consistent with hemolysis and bone marrow response.
Reticulocyte (%)	~0.5–2.5	2.8 H	N/A	10.9 H	5.4 H	6.3 H	Elevated—consistent with hemolytic response.
LDH (U/L)	<280 typical	1334 H	1027 H	271	341	N/A	Markedly elevated initially—classic hemolysis marker. Improves with treatment.
Haptoglobin (mg/dL)	30–200	<30 L	<30 L	103	53	110	Low initially (hemolysis); normalizes after treatment.
Fibrinogen (mg/dL)	200–400	384	310	N/A	N/A	N/A	Normal levels—helps rule out DIC.
PTT (sec)	~25–35	27.6	27.3	N/A	28.4	N/A	Within normal limits—no coagulopathy.
INR	~1.0	1.0	N/A	N/A	1.0	N/A	Normal—supports non-DIC etiology.
ADAMTS13 activity	>0.10	<0.03 L (Panic)	N/A	N/A	<0.03 L (Panic)	N/A	Severely deficient—diagnostic for TTP. Persistent despite initial therapy.
ADAMTS13 inhibitor	<0.4	4.5 H	N/A	N/A	3.5 H	N/A	Confirms acquired autoimmune TTP.
Vitamin B12 (pg/mL)	200–900	169 L	N/A	N/A	1476 H	N/A	Initially deficient; high after supplementation.
*H. pylori *antigen	Negative	N/A	N/A	N/A	N/A	Positive	May contribute to autoimmune activity—consider eradication therapy.
Peripheral smear	Normal	None	Slight Schistocytes	Slight	N/A	N/A	Fragmented RBCs observed—supports microangiopathy. Plasmic score of 5: Intermediate TTP risk.

**Figure 1:**
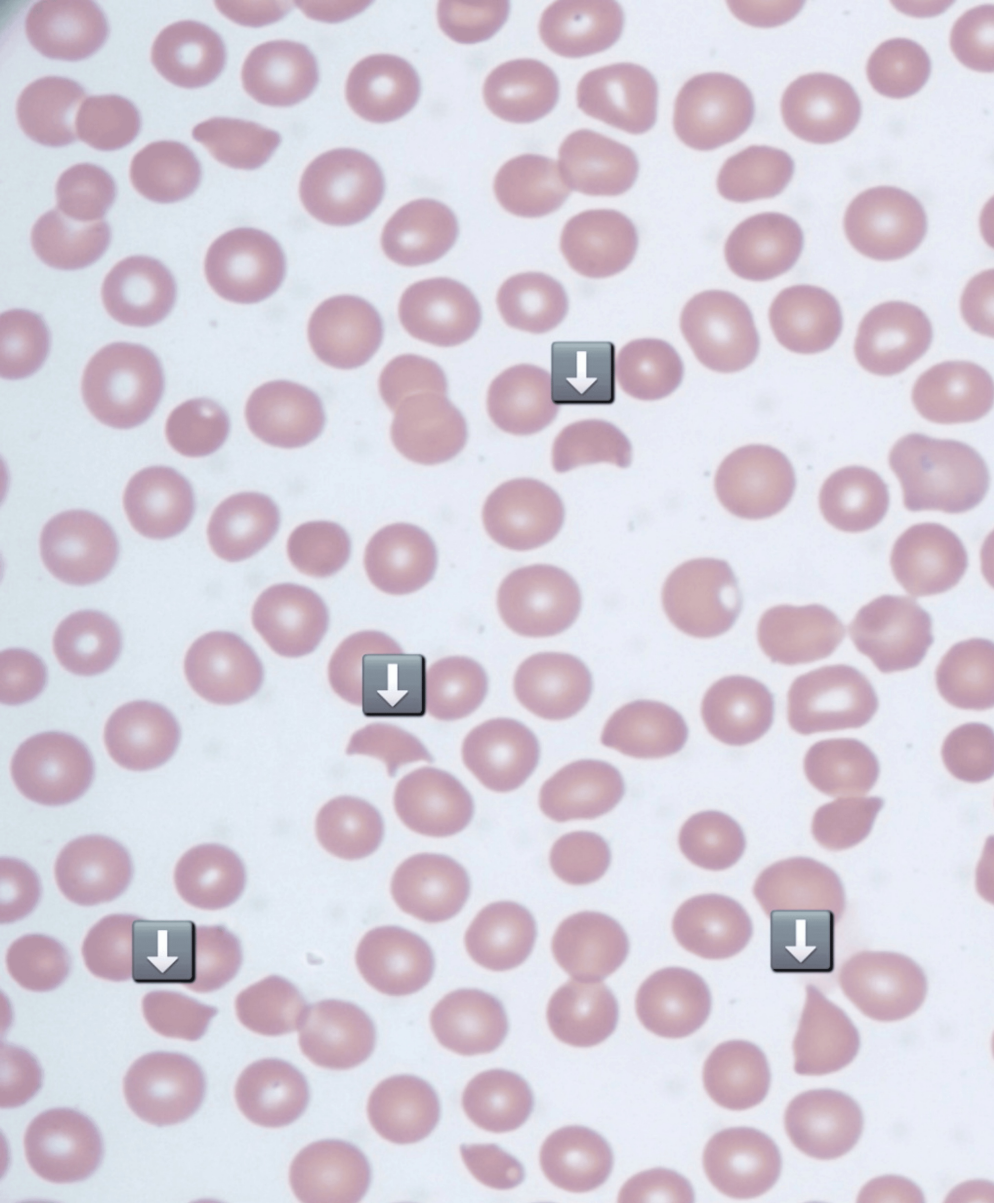
Peripheral blood smear before plasma exchange showing severe thrombocytopenia and schistocytes

The patient was initially treated with systemic corticosteroids for presumed immune thrombocytopenic purpura (ITP). Repeat laboratory evaluation after 24 hours showed the following: total bilirubin: 3.6 mg/dL, direct bilirubin: 0.5 mg/dL, LDH: 1,027 U/L, Hb: 11.0 g/dL, platelets: 11,000/µL, calcium: 8.5 mg/dL, troponin: 101 ng/L, haptoglobin: <30 mg/dL, vitamin B12: 151 pg/mL, and WBC: 13.1 ×10⁹/L. Figure 1 shows the peripheral blood smear findings after 24 hours, showing severe thrombocytopenia now with the presence of schistocytes.

**Figure 2:**
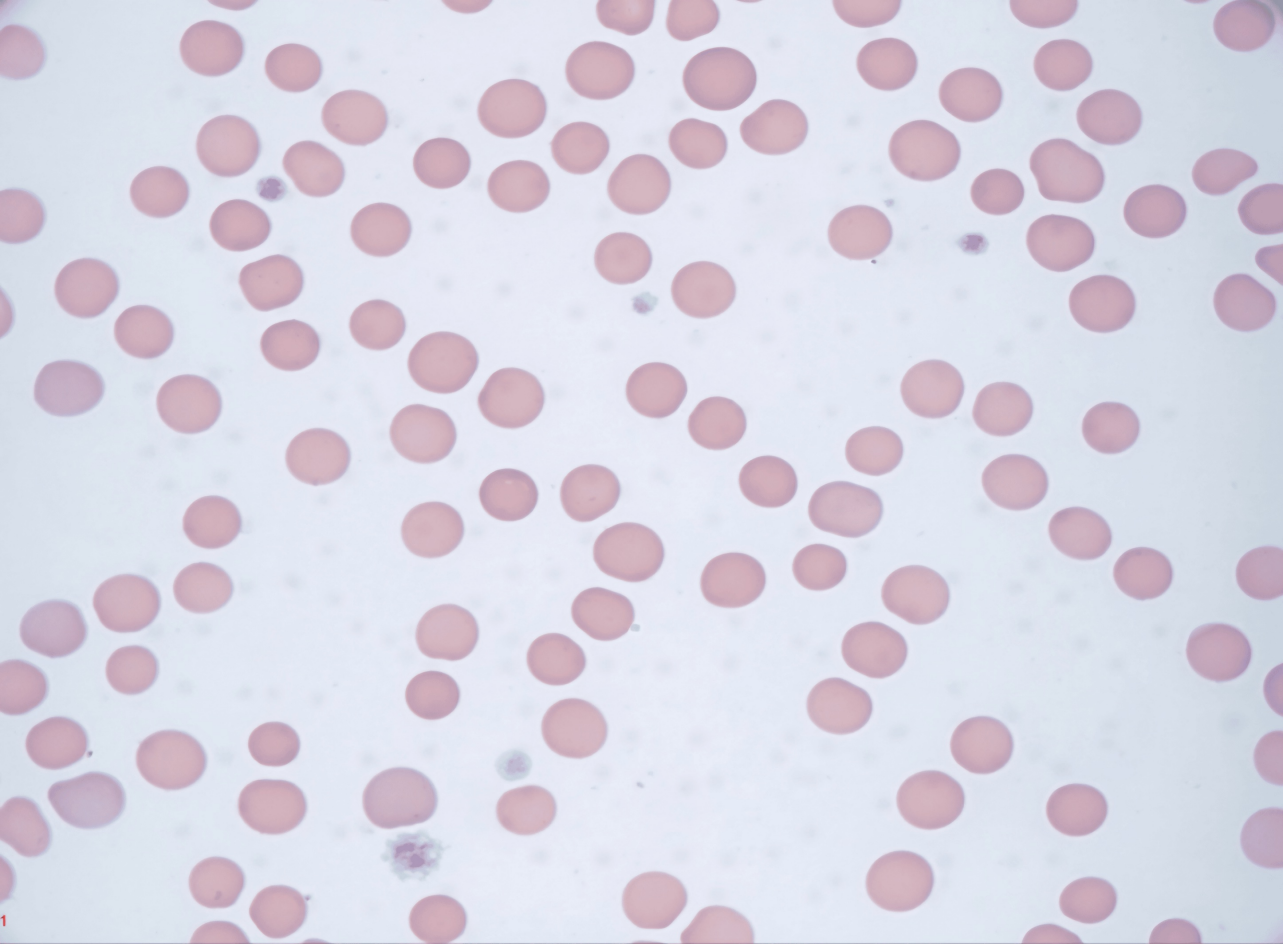
Peripheral blood smear after commencing plasma exchange showing improved thrombocytopenia and no schistocytes.

Due to the lack of response to corticosteroids, a significant drop in Hb, the appearance of schistocytes, and a PLASMIC Score of six, this was high risk for TTP. Subsequent testing confirmed the diagnosis with ADAMTS13 activity levels of <0.03 and an ADAMTS13 inhibitor level of 4.5. A femoral central venous catheter was placed, and the patient underwent therapeutic plasma exchange (TPE) for five days, receiving a total of 62 units of plasma. He was also treated with intravenous rituximab, vitamin B12 supplementation, and continued on systemic corticosteroids. The patient’s platelet count improved significantly, from 8,000/µL to 243,000/µL. Figure 2 shows the peripheral blood smear findings after commencing plasma exchange, showing improved thrombocytopenia without schistocytes.

The patient was discharged on a prednisone taper, continued vitamin B12 supplementation, and scheduled for outpatient hematology follow-up for weekly rituximab infusions over four weeks. However, he failed to attend his follow-up appointments due to persistent lethargy and financial constraints. He also ran out of prednisone one week before readmission.

Upon eventual follow-up in the hematology clinic, a repeat complete blood count revealed severe thrombocytopenia, prompting hospital readmission. During the second hospitalization, the patient responded favorably to high-dose corticosteroids. He tested positive for H. pylori, and quadruple therapy was initiated, consisting of bismuth subsalicylate, tetracycline, metronidazole, and pantoprazole.

## Discussion

TTP is characterized by platelet aggregation, erythrocyte fragmentation with associated thrombocytopenia, and organ ischemia. Deficiency of ADAMTS13, which is responsible for cleaving vWF, results in unusually large vWF multimers. There is an interaction between these ultra-long vWF multimers that spontaneously form aggregates with platelets, leading to microthrombi in the small blood vessels and to the clinical manifestations of TTP. 

iTTP is an autoimmune condition in which auto-antibodies neutralize ADAMTS13 protease activity; however, the full details of its etiology are not yet known. It is known that iTTP is associated with multiple infectious organisms, of which viruses have been primarily associated, followed by bacteria, of which *H pylori *has been mostly cited [6]. There have been multiple proposed mechanisms by which *H. pylori *can trigger, potentiate, or assist in iTTP. The proposed mechanisms include molecular mimicry, platelet aggregation, and cytokine release.

Molecular mimicry through the activation of self-reactive ADAMTS13 T cells

Many viral and bacterial infections have been associated with the development of iTTP. Of viruses, the most commonly cited associations include HIV, hepatitis C, and influenza. For bacteria, the most commonly cited associations include *H. pylori*, *Staphylococcus aureus*, and *Escherichia coli* [6]. The variety of microbial associations with iTTP suggests that there likely is an immunological association. 

Pos et al. proposed that via pattern recognition receptors, microorganisms trigger the innate immune system, resulting in up-regulation of MHC class II and costimulatory molecules and the activation of ADAMTS13-specific T cells that have escaped negative selection in the thymus [7]. After processing, the microorganism-derived peptides are loaded onto MHC class II and presented to the T-cell receptor of an auto-reactive CD4 T-cell, and through similarities with the microorganism-derived peptide, the CD4 T cells cross-react with ADAMTS13-derived peptides [6].

These observed associations of iTTP with bacterial infections have not yet been substantiated by any direct link to specific pathogen antigens resembling ADAMTS13.

*H. pylori*-induced platelet aggregation 

There are several mechanisms through which H.Pylori induces platelet aggregation, which can potentially contribute to the thrombotic events seen in iTTP. These proposed mechanisms are as follows:

H. pylori: VWF Complex Interacts With GP1b

Bryne et al. in a study examining the mechanism of interaction between* H. pylori* and platelets was able to show that three of five tested strains of *H. pylori *were able to induce platelet aggregation in the presence of VWF and anti-*H. pylori *antibodies. The proposed mechanism is that *H. pylori *binds to VWF, forming a complex that interacts with GP1b to induce platelet activation and aggregation [8].

Franchini further proposed that the aggregation of platelets triggered by the interaction between *H. pylori *and von Willebrand factor (VWF) may not be adequately regulated by the proteolytic activity of ADAMTS13, likely due to an acquired deficiency of this enzyme, thereby resulting in microvascular thrombosis [4].

H. pylori Stimulates P-Selectin Expression

Another mechanism of *H. pylori*-induced platelet aggregation is via stimulation of the expression of P-selectin [9]. Yeh et al. found that *H. pylori*-induced P-selectin release depends on the presence of specific IgG and plays a crucial role in platelet aggregation. In addition, signs of platelet apoptosis and phosphatidylserine expression were observed, suggesting that *H. pylori *may reduce platelet counts through both aggregation and apoptotic pathways [9].

H. pylori Urease 

The presence of *H. pylori urease* (HPU) allows this organism to survive in the stomach through its neutralizing effect on the acidic medium; however, it has also been found to activate platelets independently of its enzymatic function. Wassermann et al. showed that purified recombinant HPU induced platelet aggregation and ATP release in rabbits, with effects mediated by calcium channels and 12-lipoxygenase activity but not by ureolytic action or platelet-activating factor [10]. These findings indicate that HPU may contribute to the development of platelet aggregation in iTTP through a lipoxygenase-mediated pathway. 

*H. pylori* stimulates cytokine release

Takatsuka et al. investigated the association between *H. pylori* infection and the development of TTP or hemolytic uremic syndrome (HUS) in bone marrow transplant (BMT) recipients. Among 74 patients, six developed TTP/HUS. Although no significant differences in clinical characteristics were observed between groups, those who developed TTP/HUS had significantly higher *H. pylori *positivity rates (p < 0.05), as well as elevated levels of interleukin (IL)-12 and IL-8 during leukocyte recovery and at the onset of TTP/HUS (both p < 0.05)[11]. Supporting this further, there have been several reports that *H. pylori *stimulates IL-12 and IL-8 [12-14]. *H. pylori*, through stimulating pro-inflammatory cytokines such as IL-12 and IL-8, may play a role in the pathogenesis of iTTP by promoting inflammation and platelet activation [14]. 

Gringauz et al. described a case of chronic* H. pylori *infection in an 81-year-old female with TTP that was highly resistant to medical treatment and then had complete resolution of TTP following *H. pylori* infection treatment [15]. This emphasizes that iTTP can be triggered by infections including *H. pylori*, and identification and treatment may play a significant role in improving outcomes.

These multiple mechanisms highlight the complex interplay between *H. pylori* infection and the onset and recurrence of iTTP, involving both direct bacterial interactions with platelets and immune-mediated processes.

## Conclusions

The patient’s presentation of petechiae, thrombocytopenia, microangiopathic hemolytic anemia, elevated LDH, and indirect hyperbilirubinemia supported a diagnosis of TTP, typically caused by severe ADAMTS13 deficiency. This was confirmed by ADAMTS13 activity testing, prompting treatment with plasma exchange, glucocorticoids, and rituximab. In iTTP, ADAMTS13 deficiency results from circulating autoantibodies that inhibit its activity. Several mechanisms have been proposed to explain how Helicobacter pylori may contribute to iTTP, including molecular mimicry, direct platelet aggregation, and pro-inflammatory cytokine release. This case, along with others in the literature, highlights the potential role of *H. pylori* in triggering or exacerbating iTTP through immune and platelet-mediated pathways.

Given this possible association, clinicians may consider testing for and eradicating *H. pylori* in patients with iTTP, particularly in cases of recurrent or refractory disease, even in the absence of formal guidelines. Further research is needed to clarify causality and determine whether such interventions could reduce relapse rates and improve long-term outcome.
